# Bilateral tonic seizures probably induced by eperisone hydrochloride: a case report

**DOI:** 10.3389/fneur.2023.1240526

**Published:** 2023-09-14

**Authors:** Long Luo, Jun Yin, Zhigang Li, Wei Zhang, Ying Yuan, Ying Tang, Ye Deng, Ling Zhu

**Affiliations:** ^1^Department of Neurology, Xiangtan Central Hospital, Xiangtan, Hunan, China; ^2^Department of Neurology, Xiangya Hospital, Changsha, Hunan, China

**Keywords:** eperisone hydrochloride, muscle relaxant, drug, tonic, seizures, case report

## Abstract

Eperisone hydrochloride is a central muscle relaxant used to treat osteoporosis. Seizures are rare side effects of eperisone hydrochloride and have been previously reported in the medical literature in overdose situations but not at regular doses. This case report describes a 42-year-old male painter who developed severe bilateral tonic seizures after the initiation of eperisone hydrochloride at regular doses for low back pain. Symptoms gradually eased in the days following the discontinuation of eperisone hydrochloride and antiepileptic treatment, with no recurrence. This rare adverse drug reaction warrants clinical awareness; however, the mechanisms underlying these adverse reactions remain to be clarified.

## Highlights

- Seizures are rare side effects of eperisone hydrochloride.
- Seizures may be induced by either overdose or routine amounts of eperisone hydrochloride.
- Antiepileptic therapy is an effective treatment for eperisone hydrochloride induced seizures.

## Introduction

A sustained increase in muscle contraction, lasting from a few seconds to minutes, is known as a tonic (1). Awareness is the ability of individuals to perceive their surrounding environment and their own state and is dependent on the excitation of the cerebral cortex (2). Bilateral tonic seizures manifested as a bilateral simultaneous onset of tonic with impaired awareness is a generalized seizure classification, and a prior study estimated that approximately 6% of new-onset seizures are drug-induced (3).

Eperisone hydrochloride is a central muscle relaxant widely used in most Asian countries for treating muscle tension [i.e., cervical spondylosis (4) and low back pain (5)] and spastic paralysis (6). Eperisone hydrochloride acts on the spinal cord and supraspinal structures to reduce the α- and γ-efferent activities of spinal cord neurons, thereby reducing skeletal muscle tone (7). Eperisone hydrochloride inhibits presynaptic primary afferent terminal transmitter release through a combined action on voltage-gated sodium and calcium channels (8). It also dilates blood vessels by blocking voltage-dependent calcium channels (9) and improves muscle blood flow in the trunk muscles (10). Eperisone hydrochloride blocks postjunctional α1- and α2-adrenergic, muscarinic, and serotonergic receptors, as well as prejunctional α2 adrenoceptors, and reduces PGI2 synthesis *via* mechanisms other than cyclooxygenase inhibition (11). In animal experiments, eperisone hydrochloride has been shown to block nicotinic receptors in the parasympathetic ganglia, sympathetic nerve endings, and muscarinic receptors on cardiac effector cells (12). Generalized tonic-clonic seizures (GTCS), tonic seizures, myoclonic seizures, and epileptic spasms seizures have been reported in a few cases and have been associated with an overdose of eperisone hydrochloride (13, 14), and the pathophysiology underlying the development of these symptoms has not been clarified. Here, we describe the case of a 42-year-old man who developed bilateral tonic seizures after receiving regular doses of eperisone hydrochloride for low back pain.

## Case presentation

A 42-year-old male painter was prescribed eperisone hydrochloride 50 mg tid by his local doctor for lower back pain. The patient had no significant abnormal reaction after taking eperisone hydrochloride for the first 3 days. However, on day 4, the patient took eperisone hydrochloride at 9:00 am and, at approximately 10:30 am, developed a bilateral tonic seizure accompanied by head rotation to the right without eyes turning up, incontinence, or trismus, lasting from 6–7 s to 2 min at a time and could not be aroused. Subsequently, the patient awakened and was fatigued, could not recall events during the seizure, and did not describe any aura. The symptoms reappeared after a pause of minutes to tens of minutes. The next morning, the patient was admitted to the neurology department, and on examination, the recurrent episodes recurred. During the interictal period, the patient was aware (Glasgow Coma Scale score of 15), with a sensitive pupil reflex, normal muscle strength, and tone in all four limbs, had no baroreflex signs, oral mucous membrane bites, body abrasions or bruises, and the patient's cardiovascular, respiratory, and abdominal examinations were unremarkable. The patient's vital signs were as follows: a body temperature of 36.2°C, blood pressure of 148/103 mmHg, heart rate of 125 beats/min, oxygen saturation of 96% room air, and blood sugar level of 9.3 mmol/L. An electrocardiogram revealed sinus tachycardia (122 beats/min); the plain head computed tomography (CT) scan was normal. The patient denied a history of epilepsy or any recent history of fever, upper respiratory tract infection, urinary symptoms, diarrhea, trauma, or travel. The patient had never taken eperisone hydrochloride or its analogs before and had no history of taking other concomitant drugs. The systematic review was unremarkable, except for hepatitis B positivity.

**Figure 1:**
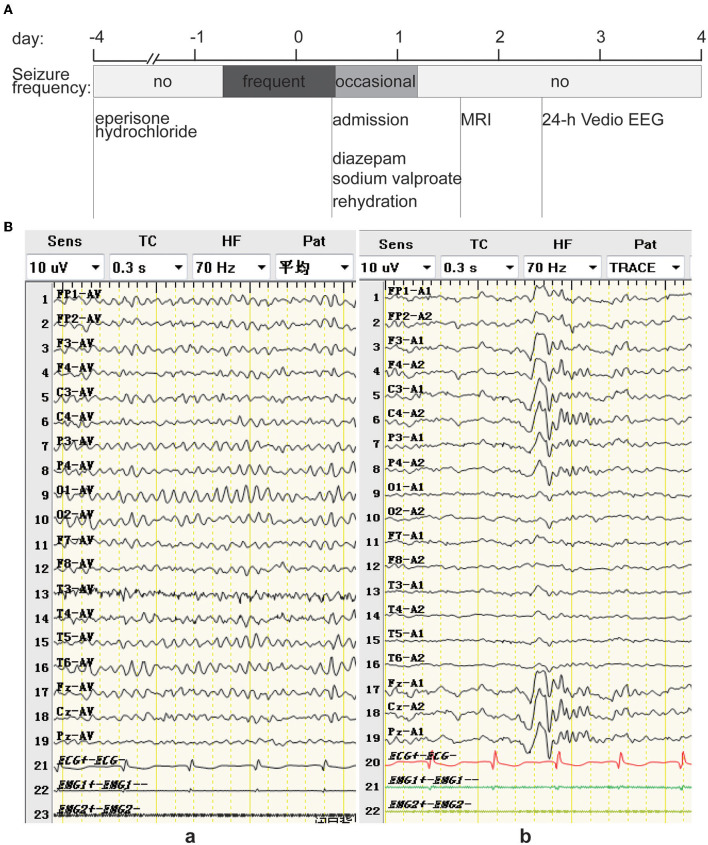
**(A)** Timeline of an episode of care. The patient ingested 50 mg tid of eperisone hydrochloride for the first 3 days with no adverse effects. However, on the morning of the 4th day, the patient experienced frequent bilateral tonic seizures. The following day, the patient was admitted to the hospital and treated with diazepam, sodium valproate, and rehydration, after which the symptoms decreased and did not occur on the morning of the 2nd day after admission. The head MRI was normal on the 2nd day after admission, sodium valproate was discontinued on the 3rd day after admission, and the 24-h video EEG from the 3rd to the 4th day after admission was normal. **(B)** EEG showing low-to-medium amplitude alpha background EEG predominantly at 8.5–11.5 Hz in the quiet, awake with closed-eye state (a), and K complex waves during sleep (b).

The patient's laboratory test results after admission were as follows: platelet count, 358 × 10^9^/L (normal range: 85–303 × 10^9^/L); prothrombin time activity, 132% (normal range: 75–130%); urea level, 2.6 mmol/L (normal range: 2.9–8.2 mmol/L); HBV-DNA, 1.021 × 10^2^ IU/mL (normal range: < 20 IU/mL); HBsAg, 95.830 ug/L (normal range: < 0.05 ug/L), HBeAb, 49.317 U/mL (normal range: ≤ 0.77 U/mL); and HBcAb, 370.774 U/mL (normal range: ≤ 4.58 U/mL). The HBsAb, HBeAg, anti-HCV antibody, HIV antigen-antibody, treponema pallidum-specific antibody, alpha-fetoprotein, and abnormal prothrombin levels were normal. Blood gas, routine urine, routine fecal, hepatic function, muscle enzyme, electrolyte, blood lipid, D-dimer, thyroid function, glycated hemoglobin, and coronavirus disease 2019 nucleic acid test results were normal. The head magnetic resonance imaging (MRI) and 24-h video electroencephalogram (EEG) (Figure 1B) were normal.

Considering the patient's recurrent bilateral tonic seizures and that he was treated with diazepam (10 mg IV), sodium valproate was intravenously infused slowly at a dose of 15 mg/kg over 5 min, followed by a continuous drip at 1 mg/kg/h, and rehydration, after which the patient's symptoms decreased to once every few hours and disappeared on the morning of the 2nd day after admission. No other explanatory etiology was found in the patient's systematic review, current medical history, or laboratory tests refined after admission, and the onset of these episodes coincided with the initiation of eperisone hydrochloride. Therefore, he was diagnosed with an acute symptomatic seizure induced by eperisone hydrochloride, with a half-life of 1.6–1.8 h. After eperisone hydrochloride discontinuation for more than five half-lives and his symptoms being effectively controlled, sodium valproate was discontinued on the 3rd day after admission, and the patient recovered and was discharged within 4 days without any adverse sequelae (Figure 1A). There was no disease recurrence during the follow-up period of approximately 1 year.

The reporting of this study conforms to the CARE guidelines (15).

## Discussion

**Figure 2:**
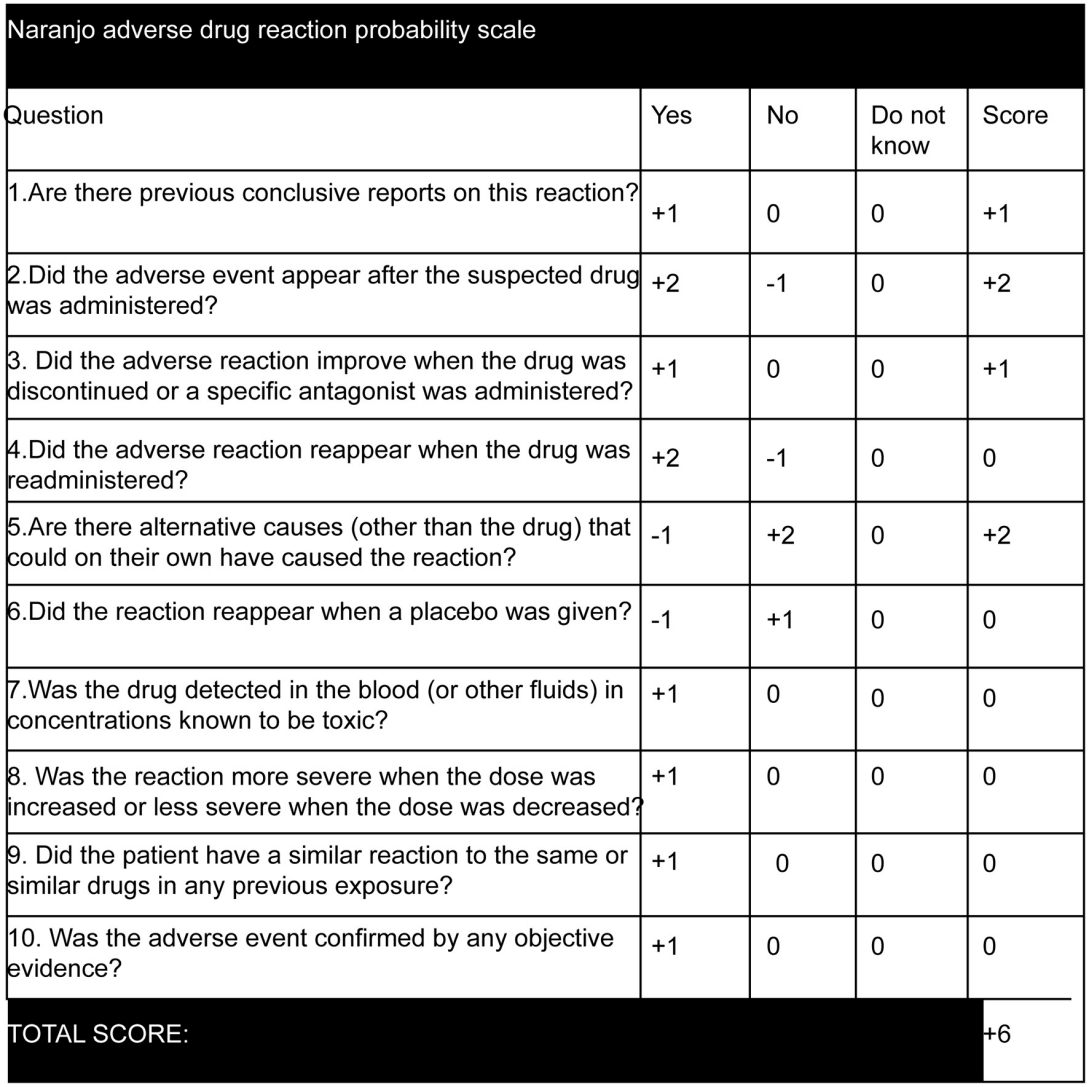
Naranjo adverse drug reaction probability scale (16). The score was calculated as +6 as eperisone hydrochloride has been previously reported to cause tonic seizures, myoclonic seizures, and epileptic spasms seizures, with aware or impaired awareness (14) and GTCS (13). The adverse event occurred after oral administration of eperisone hydrochloride and symptoms improved after discontinuing the drug. Other causes for bilateral tonic seizures were ruled out.

There is limited information on eperisone hydrochloride causing seizures. According to a Japan Poison Information Center survey conducted between 2001 and 2010, five patients developed motor seizures due to the overdose of eperisone hydrochloride, which manifested as tonic seizures, myoclonic seizures, and epileptic spasms seizures, and three patients showed impaired awareness (14). In addition, GTCS has been reported in infants who have overdosed on eperisone hydrochloride (13). Our report presents a case of bilateral tonic seizures in the context of conventional doses of eperisone hydrochloride. The calculation of the Naranjo adverse drug reaction probability scale (16) for our patient (represented in Figure 2) yielded a score of +6, which determined a “probable” drug-related cause for the associated symptoms. The severity score on the modified Hartwig and Siegel scale (17) was level 5 as the patient required hospitalization for 4 days due to a serious adverse reaction.

In this case, the patient did not experience a side effect until the 4th day of drug administration, which may have been a delayed drug reaction as there is no accumulation of the drug in healthy adult males (18). Although the exact cause of the delayed drug reactions is unknown, such delayed reactions to drugs occasionally occur in clinical practice, such as drug-induced delayed hypersensitivity reactions (19). However, eperisone hydrochloride is mainly biotransformed in humans by carbonyl reduction and hydroxylation (20). Hepatitis B has negative effects on carbonyl reduction (21). Therefore, it cannot be ruled out that the patient in this case had an abnormal eperisone hydrochloride metabolism. Although most drug-induced seizures are self-limiting, prolonged recurrent seizures can lead to serious complications that need to be managed, and benzodiazepines are the first-line anticonvulsants for the treatment of drug-induced seizures (22). Furthermore, although there has been limited experience with valproic acid in the treatment of drug-induced epilepsy, valproic acid pretreatment has been found to increase the central nervous system threshold for theophylline-induced seizures in rats (23) and effectively prevent clozapine-induced seizures (24). In patients with acute symptomatic epilepsy, antiepileptic treatment limited to 7 days is reasonable if the duration is sufficient to correct the cause (25). Fortunately, the patient in this case received an intravenous push of diazepam followed by a continuous drip of sodium valproate, which effectively controlled his symptoms without recurrence.

## Limitation

This study has several limitations. First, eperisone hydrochloride was not re-challenged due to ethical concerns. Second, we could not determine the blood drug concentration of the patient due to the limitations of the local medical conditions. Third, we failed to perfect the video EEG on the patient during the seizure period. Fourth, the seizures were probably induced by eperisone hydrochloride, and long-term follow-up observations are needed to rule out other etiologies.

## Conclusion

This case report shows that oral eperisone hydrochloride, taken at regular doses, may induce bilateral tonic seizures, even though eperisone hydrochloride is considered a neurodepressant drug. We hope to raise awareness among healthcare professionals to ensure timely recognition and appropriate management of such cases. Further studies are warranted to explore the underlying mechanisms and confirm the causal relationship between eperisone and seizures.

## Data availability statement

The raw data supporting the conclusions of this article will be made available by the authors, without undue reservation.

## Ethics statement

Ethical review and approval was not required for the study on human participants in accordance with the local legislation and institutional requirements. Written informed consent from the patients/participants or patients/participants' legal guardian/next of kin was not required to participate in this study in accordance with the national legislation and the institutional requirements. Written informed consent was obtained from the individual(s) for the publication of any potentially identifiable images or data included in this article.

## Author contributions

LL, JY, and LZ: drafting the manuscript, editing the manuscript, approving the manuscript, and being accountable for manuscript integrity. ZL, WZ, YY, YT, and YD: project conception, editing the manuscript, and approving the manuscript. All authors contributed to the article and approved the submitted version.
